# Use of the Combination Therapy of Recombinant Feline Interferon-Omega Injection and Oral Prednisolone for Canine Aural Hematoma: A Retrospective Case Series

**DOI:** 10.3390/vetsci13090937

**Published:** 2026-09-10

**Authors:** Tomoya Furujo, Masayoshi Nagakawa, Kazumi Shimada, Midori Tanaka, Tomoyuki Tezuka, Daisuke Ito, Takashi Tanaka

**Affiliations:** 1 Animal Medical Center, Tokyo University of Agriculture and Technology, Tokyo 183-8509, Japan; ft2364@go.tuat.ac.jp (T.F.);; 2 Takashimadaira Tezuka Animal Hospital, Tokyo 175-0082, Japan; 3 Department of Small Animal Clinical Sciences, Texas A&M University, College Station, TX 77843, USA

**Keywords:** aural hematoma, interferon-omega, dog, prednisolone

## Abstract

This retrospective study described clinical outcomes of combining local recombinant feline interferon-omega (rFeIFN-ω) injection with oral prednisolone in the management of canine aural hematoma. The medical records of dogs presented to a private veterinary hospital between January 2019 and January 2026 were reviewed. Thirty-one dogs underwent the treatment protocol, of which five were lost to follow-up, leaving a final cohort of 26 dogs. The protocol involved a local intralesional injection of 10.0 MU of rFeIFN-ω without fluid evacuation alongside concurrent oral prednisolone (1.0 mg/kg once daily) without anesthesia at 1-week intervals. Complete initial resolution was achieved in 21 dogs (80.8%) with a mean of 2.37 ± 1.14 treatment sessions, whereas 5 dogs discontinued therapy prematurely prior to complete resolution because the owners declined further treatment visits, leaving their final outcomes undetermined. Recurrence was documented in five of the 21 complete resolution cases (23.8%), spanning from 2 weeks to 1 year post-resolution. Two of these dogs underwent re-treatment and achieved complete clinical resolution during the available follow-up period. No clinically relevant adverse effects occurred. While the independent therapeutic efficacy of rFeIFN-ω remains unproven, these preliminary findings suggest that the combination protocol may represent a feasible, non-surgical outpatient option for managing canine aural hematoma.

## Simple Summary

Aural hematoma is a frequently encountered condition in dogs characterized by a fluid-filled swelling within the ear flap. Because the exact cause remains unclear, no definitive standard treatment exists, and existing options often involve surgical intervention under anesthesia or repeated needle drainage. This study investigated a novel non-surgical option using a combination of a localized injection of an antiviral protein (recombinant feline interferon-omega) into the ear flap and concurrent oral steroid medication, administered without any anesthesia or sedation. Among the 26 dogs analyzed, every case showed clinical improvement, and 80.8% achieved complete resolution with a mean of 2.37 ± 1.14 treatment sessions. Although recurrence was observed in 23.8% of cases within a year, the dogs that underwent re-treatment achieved successful re-healing. No significant adverse effects were recorded. This non-surgical combination protocol may represent a feasible, minimally invasive treatment option for canine aural hematoma without requiring general anesthesia or sedation. This work provides preliminary clinical information on a minimally invasive outpatient treatment approach that may be useful when avoidance of general anesthesia or sedation is desirable.

## 1. Introduction

Canine aural hematoma is a frequently encountered condition in clinical practice, although its exact prevalence remains unknown [1,2,3]. It is characterized by a fluctuant swelling filled with serosanguinous exudate beneath the skin on the concave surface of the pinna [1,2,4]. The primary source of hemorrhage remains unclear. It has been hypothesized that it arises from branches of the great auricular artery and vein located within, beneath, or between the cartilage layers [1,2,5]. This condition is triggered by shearing forces generated by violent head shaking or ear scratching and is often associated with hypersensitive skin diseases, otitis externa, and ear mite infestations [1,5]. Other hypotheses include trauma, degenerative processes leading to tearing of the auricular cartilage, and inflammatory, immune-mediated processes that damage the cartilage [4,6,7]. Complicating the understanding of this pathology is the view that aural hematoma in dogs may not be a true hematoma, but rather a serosanguinous exudate that accumulates over time [4]. Because the precise pathophysiology remains incompletely elucidated, various treatments have been attempted; however, no definitive gold standard treatment has been established to date [3,5,7,8,9,10,11]. The therapeutic goals for managing aural hematoma include identifying and treating any underlying disease to prevent recurrence, establishing adequate drainage, maintaining tissue apposition, and preserving the normal appearance of the ear to avoid excessive thickening or scar formation.

Interferons are cytokines that possess a wide range of biological activities, highlighting their significant potential as therapeutic agents [12,13]. Their documented in vivo functions include immunoregulation, anti-inflammatory effects, and antitumor activities [12,13]. Recombinant feline interferon-omega (rFeIFN-ω; INTERCAT, Toray Industries, Inc., Tokyo, Japan) was first developed and marketed in 1994 as an antiviral drug for feline calicivirus infection [14]. To date, rFeIFN-ω has demonstrated efficacy not only against feline viral infection but also against canine parvovirus infection [13,15,16,17,18,19,20]. Furthermore, in vitro studies have reported that rFeIFN-ω exerts antiproliferative effects on certain canine and feline tumor cells [13,21,22,23]. These findings suggest that its therapeutic potential extends well beyond the treatment of infectious diseases, promising diverse clinical applications.

Although the detailed pathophysiology of canine aural hematoma remains unclear, anti-inflammatory therapy is considered effective [5,10,24]. Therefore, the local administration of rFeIFN-ω, which possesses anti-inflammatory properties, may offer a new approach to treating this condition, and rFeIFN-ω is currently being used off-label for the treatment canine aural hematoma in some clinical facilities in Japan. This treatment protocol involves the local injection of rFeIFN-ω into the hematoma site combined with concurrent oral prednisolone. However, to date, no studies have investigated the therapeutic efficacy of rFeIFN-ω for canine aural hematoma. Therefore, the purpose of this retrospective study was to describe clinical outcomes following this combination protocol in dogs with aural hematoma treated at a single private animal hospital.

## 2. Case Presentation

### 2.1. Ethics Statement

When a diagnosis of aural hematoma was established, conventional therapeutic options, including surgical intervention and needle aspiration, were fully presented to the owners prior to discussing the alternative regimen. Subsequently, pet owners were thoroughly informed that the local administration of rFeIFN-ω constituted an off-label (non-approved) application for this condition. Treatment was exclusively initiated only after the owners selected this option and provided explicit informed consent.

### 2.2. Animal and Data Collection

This retrospective study reviewed dogs with aural hematoma at a single private veterinary hospital (Takashimadaira Tezuka Animal Hospital) in Japan between January 2019 and January 2026. Initially, a total of 40 dogs diagnosed with aural hematoma were extracted from the electronic medical record system. Among these, 9 received alternative therapeutic regimens and inconsistent treatment protocol, and were excluded from this study. These alternative regimens included: needle aspiration alone, needle aspiration combined with oral prednisolone, oral prednisolone, rFeIFN-ω (10.0 MU) monotherapy without prednisolone, low-dose rFeIFN-ω (5.0 MU) with oral prednisolone, needle aspiration with ear taping, or no active treatment. The remaining 31 dogs initiated the study treatment protocol. Among these 31 dogs, 5 were lost to follow-up after the initial treatment session and were subsequently excluded. Consequently, a final analytical cohort of 26 dogs was evaluated in this study. For each case, the following clinical data were collected and evaluated: age at onset, sex, breed, presence of concurrent otitis externa, total number of treatment sessions, treatment success rate, recurrence, and adverse effects. The exclusion criteria consisted of cases that were lost to follow-up during the treatment course, and cases in which a treatment protocol different from those described below was administered. Regarding the clinical outcomes, complete resolution was defined as the visual and palpation-based assessment where the thickness and appearance of the affected pinna returned to a level comparable to the contralateral unaffected pinna, with no palpable fluid remaining. Clinical improvement was defined as an observed reduction in fluid accumulation within the hematoma cavity compared to baseline, without achieving complete resolution. These clinical evaluations were performed at each weekly visit through routine physical examination by attending veterinarians; objective tools such as caliper measurements, ultrasonography, or standardized volumetric scoring were not utilized.

### 2.3. Treatment Protocol

**Figure 1:**
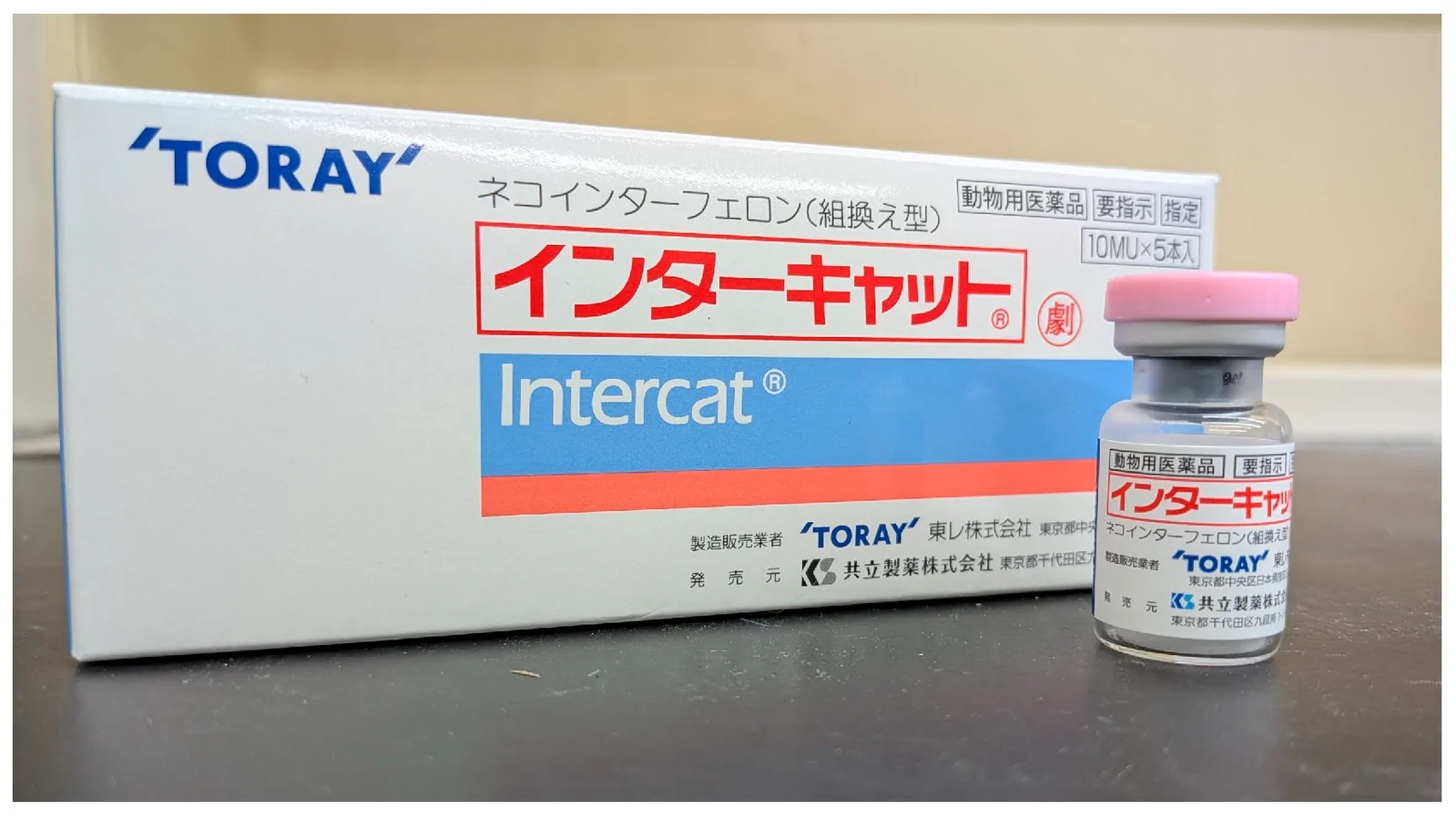
Commercial formulation of recombinant feline interferon-omega (Intercat; Toray Industries, Inc., Tokyo, Japan) utilized in the therapy for feline and canine infectious diseases.

Commercially available rFeIFN-ω (Intercat; Toray Industries, Inc., Tokyo, Japan; Figure 1) was reconstituted in 1 mL of sterile saline according to the package insert. The affected pinnal surface of each dog was aseptically prepared using iodine and alcohol. Without any general anesthesia or sedation, rFeIFN-ω was administered locally into the fluctuant swelling of the aural hematoma at a dose of 1.0 mL (10.0 MU) using a 28-gauge Myjector syringe. Crucially, the serosanguinous hematoma fluid was not aspirated or evacuated prior to or during the injection procedure. The fixed dose of 10.0 MU was applied uniformly across all dogs, regardless of body weight, breed, or hematoma size. Immediately following the injection, digital pressure was applied to the puncture site for several minutes to prevent leakage of the intralesional fluid. Concurrently, all dogs were prescribed oral prednisolone at an initial dose of 1.0 mg/kg once daily. Oral prednisolone was continued at this daily dosage throughout the treatment period and was tapered off only after achieving complete resolution or protocol termination. No additional systemic or topical medications, such as otic preparations, gastroprotective agents, or other anti-inflammatory drugs, were administered during the study period. The clinical status of the aural hematoma was reassessed at 1-week intervals. If complete resolution was not achieved, repeat local administrations of rFeIFN-ω were performed in the same manner described above.

### 2.4. Data Analysis

Descriptive statistics were used to summarize the clinical data. Quantitative variables, including age and the number of treatment sessions, were expressed as median (with ranges) or means ± standard deviations, as appropriate. Qualitative variables, such as sex, breed, and the presence of concurrent otitis externa, were presented as percentages. No formal comparative statistical analysis or hypothesis testing were performed due to the descriptive nature and sample size of this study. Exact 95% confidence intervals were calculated using the Clopper-Pearson method. All data analyses were performed using GraphPad Prism version 11.0 (GraphPad Software, San Diego, CA, USA).

### 2.5. Case Characteristics

A total of 31 dogs underwent the treatment protocol during the study period. Of these, 5 dogs were lost to follow-up during the treatment course and consequently excluded, resulting in a final analytical cohort of 26 dogs (Table S1). The baseline demographic and clinical characteristics of these 26 dogs are summarized in Table 1. The median age at presentation was 9 years (range, 1–14 years). The study population included 15 females (57.7%; 7 intact and 8 spayed) and 11 males (42.3%; 6 intact and 5 castrated). In terms of breed distribution, the most frequently affected breeds were French Bulldogs (*n* = 5), followed by Golden Retrievers (*n* = 4) and Labrador Retrievers (*n* = 4). The remaining cohort comprised one representative each of the following breeds (*n* = 1 each): Beagle, Weimaraner, American Cocker Spaniel, Welsh Corgi, Standard Poodle, Toy Poodle, West Highland White Terrier, Shiba Inu, Shetland Sheepdog, German Shepherd, Maltese, Miniature Pinscher, and Mixed-breed. Of the 26 dogs, 11 dogs (42.3%) presented with concurrent otitis externa that required active therapeutic intervention. The diagnosis of otitis externa was established based on history and clinical signs (e.g., aural pruritus, head shaking, or ear discharge) combined with otoscopic examination demonstrating erythema or swelling. These 11 dogs were not under active treatment for chronic otitis prior to presentation; rather, otitis externa was identified concurrently upon presentation for the aural hematoma. Regarding the management of otitis externa, in-clinic ear flushing with sterile saline was performed during the initial visit prior to the rFeIFN-ω injection. No topical otic medications (e.g., antimicrobial or anti-inflammatory ear drops) were prescribed for home use; management relied solely on the systemic effect of the prescribed oral prednisolone.

### 2.6. Clinical Outcomes

**Figure 2:**
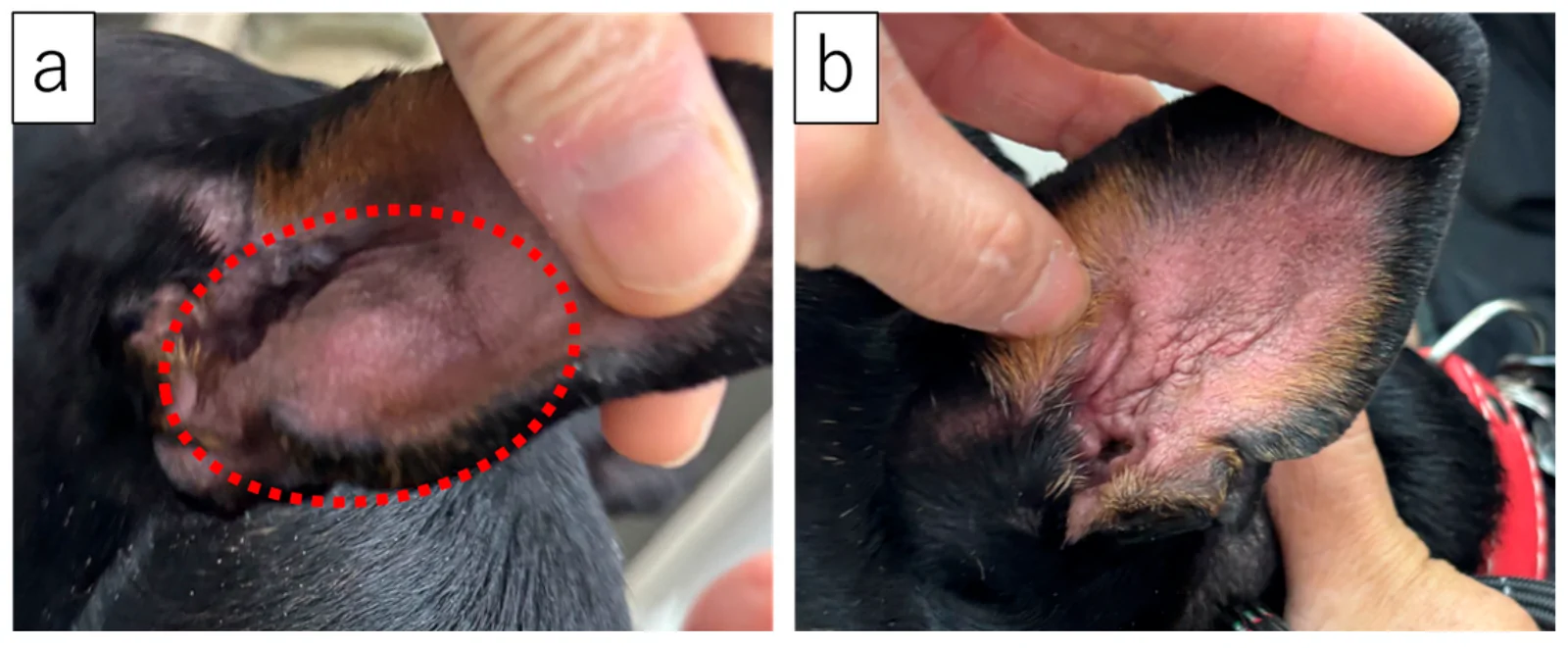
Representative clinical responses of a canine aural hematoma treated with the combination therapy of recombinant feline interferon-omega and prednisolone: (**a**) Appearance at initial presentation (before treatment), exhibiting swelling of the pinna; (**b**) appearance after three treatment sessions (after treatment). The fluid-filled swelling caused by the aural hematoma was completely resolved. The dashed line delineates the swollen area of the aural hematoma.

The representative clinical appearance of a case before and after the combination therapy was illustrated in Figure 2 (Case 9). A comparison between before and after the treatment demonstrated a marked reduction in the fluid-filled swelling of the pinna, confirming the successful preservation of the normal physical structure of the ear flap without severe deformity.

The clinical outcomes and treatment-related data are summarized in Table 2. The mean number of treatment sessions was 2.37 ± 1.14, with a range of 1–5 sessions. Clinical improvement was recognized in all cases (26/26, 100%; 95% CI: 86.8–100.0%). Among them, complete initial resolution during the study period was achieved in 21 out of the 26 dogs (80.8%; 95% CI, 60.7–93.5%). Specifically, 3 dogs (14.3%) resolved after 1 session, 11 dogs (52.4%) after 2 sessions, 4 dogs (19.0%) after 3 sessions, 1 dog (4.8%) after 4 sessions, and 2 dogs (9.5%) required 5 sessions. The remaining 5 dogs that demonstrated initial clinical improvement did not achieve complete resolution because the owners elected to decline further weekly treatment sessions (e.g., satisfaction with partial swelling reduction or reluctance for repeated clinical visits). Because the treatment protocol was not completed, the ultimate long-term outcomes for these 5 cases remain undetermined.

Among the 21 dogs that achieved complete initial resolution, recurrence was documented in 5 cases (23.8%; 95% CI: 8.2–47.2%). The time to recurrence for each of these 5 cases was 2 weeks, 1 month, 2 months, 6 months, and 1 year following the complete resolution of the initial episode, respectively. Out of these 5 recurrent cases, 2 dogs underwent re-treatment using the combination treatment of rFeIFN-ω and oral prednisolone, while the owners of the remaining 3 dogs declined further treatment, resulting in these cases being lost to long-term follow-up. Notably, both of the re-treated dogs achieved complete and permanent clinical remission, requiring 1 and 2 additional treatment sessions, respectively. No clinically relevant systemic or localized adverse effects were recorded in any of the cases. However, routine hematological or biochemical follow-up evaluations were not performed post-treatment.

## 3. Discussion

To the best of our knowledge, this is the first report describing clinical outcomes following intralesional rFeIFN-ω injection combined with oral prednisolone for canine aural hematoma. In this study, clinical improvement was achieved in 26 out of 26 dogs (100%), and complete initial resolution was successfully achieved in 21 dogs (80.8%) (Table 2). Although the remaining 5 dogs showed partial clinical improvement, they discontinued the protocol prior to complete resolution because the owners declined additional treatment sessions. While these cases cannot be classified as confirmed therapeutic failures, their final clinical outcomes remain undermined due to the incomplete protocol. These preliminary clinical observations suggest that this combination protocol may represent a feasible non-surgical management option for canine aural hematoma.

Studies investigating the specific treatment modalities selected for canine aural hematoma remain limited [3,5]. A previous report from the UK indicated that needle aspiration is the most frequently performed initial intervention, although surgical intervention is classically recommended [3]. However, conventional surgical interventions carry documented limitations, including postoperative pinnal deformity, notable recurrence rates, and inherent risks associated with general anesthesia or sedation [3,5,10]. Recently, the surgical technique of continuous vacuum drainage has been reported, demonstrating a high cure rate with excellent cosmetic outcomes [9]. Nevertheless, this approach still mandates general anesthesia and requires specialized equipment, precluding it from becoming the gold standard treatment. Conversely, as non-surgical options that circumvent anesthesia, several protocols have been documented with varying success rates, including oral prednisolone monotherapy (80%), drainage combined with local corticosteroid instillation (43%), and intra-lesional platelet-rich plasma (PRP) injection (100%) [7,10,11,24]. While these non-surgical approaches offer distinct procedural advantages, direct comparisons of therapeutic efficacy across studies are precluded by differences in study designs, sample size, patient populations, and outcome definitions. In this study, our observed initial complete resolution rate of 80.8% suggests that the combination protocol of intralesional rFeIFN-ω and oral prednisolone represents another feasible non-surgical option that can be routinely performed in an outpatient setting without anesthesia or sedation.

The treatment protocol implemented in this study offers a valuable advantage in terms of its procedural simplicity. Because rFeIFN-ω is a commercially available product, it is easily accessible for clinical practice. Furthermore, the pre-administration preparation is minimal, requiring only the reconstitution of the product according to the manufacturer’s recommendations. The procedure itself can be performed safely without anesthesia or sedation; it simply involves a local injection into the fluctuant swelling of the aural hematoma using a fine-gauge needle. The exceptional simplicity of both the preparation and the procedure, combined with the avoidance of anesthesia or sedation, makes this approach an attractive and viable alternative for patients with high anesthetic risks.

In the present study, the combination protocol required a mean of 2.37 + 1.14 treatment sessions to achieve initial complete resolution (Table 2). Characteristically, this procedure delivers rFeIFN-ω locally without prior evacuation of the hematoma fluid. Although this approach induced a minor, transient leakage of intralesional fluid at the puncture site, it caused no immediate gross changes in the conformational swelling or architecture of the pinna immediately post-injection. In responsive cases, however, a progressive, time-dependent regression of the pinnal swelling was observed. Importantly, the number of treatment sessions—and consequently the overall time required for resolution—observed in the present study was comparable to those reported in previous literature [10,11]. As a single administration rarely yields complete resolution, it is paramount that clinicians manage expectations and ensure thorough pre-treatment communication with clients regarding the necessity of multiple interventions.

Currently, there is a lack of scientific data establishing the optimal dosage of rFeIFN-ω for aural hematoma. The dose of 10.0 MU implemented in the present study corresponds to exactly one vial of the commercially available product, and it is inferred that this dosage was selected primarily for its procedural ease of preparation. Consequently, future dedicated dose-finding studies are highly warranted. However, because no dose-finding study was performed, the optimal dose remains unknown and should be investigated in future prospective studies.

Regarding the treatment schedule, local administration of rFeIFN-ω was performed at 1-week intervals in all cases in this study. To date, there is no definitive scientific evidence validating that a 1-week interval is the optimal dosing schedule for this specific condition; rather, this regimen appears to have been adopted based on empirical veterinary clinical practices. It is conceivable that shortening the dosing interval might accelerate the therapeutic responses and expedite overall resolution. Consequently, along with the optimal dosage, future chronological studies are warranted to rigorously evaluate the impact of different dosing intervals on both therapeutic efficacy and clinical outcomes. Nevertheless, the clinical outcomes of the present study suggest that, in this cohort, a 1-week treatment interval was feasible protocol for the management of canine aural hematoma.

In the present study, recurrence was documented in 5 out of the 21 completely resolved cases (23.8%) (Table 2). Of these, 2 dogs achieved complete resolution following re-treatment, whereas the remaining 3 dogs were lost to follow-up due to the owner’s preference for no further intervention. To effectively prevent recurrence, concurrent management of underlying primary diseases is critical [5]; however, among the 5 recurrent cases in our cohort, only a single dog was affected by concurrent otitis externa. This suggests other mechanical or degenerative factors might influence late-onset events. Notably, the recurrence rate observed in this study is higher than that previously reported for intra-lesional PRP therapy (0%) [10], which could represent a potential limitation or disadvantage of the present combination protocol. Furthermore, the time to recurrence was highly variable among individual cases, ranging from as early as 2 weeks to as late as 1 year post-resolution. In addition, a major limitation regarding our recurrence evaluation is the lack of a standardized post-resolution follow-up protocol. Because of the retrospective nature of this medical record review, dogs that achieved complete resolution were not routinely scheduled for long-term periodic re-examinations unless owners observed new clinical signs. Consequently, exact long-term follow-up durations for non-recurrent cases could not be uniformly established, which may affect the precision of the observed recurrence rate. This lack of chronological uniformity, coupled with the clinically relevant recurrence rate, underscores the absolute necessity for clinicians to provide thorough pre-treatment communication and realistic expectations to owners when implementing this therapy.

Although the exact mechanism of action underlying the combination therapy of local rFeIFN-ω injection and oral prednisolone remains unclear, its efficacy may be hypothesized to involve combined anti-inflammatory and antiproliferative effects. Previous studies have suggested that both oral prednisolone and local PRP injections promote the healing of aural hematomas through their anti-inflammatory pathways [10,11,24]. Because rFeIFN-ω has been reported to exert anti-inflammatory effects in dogs with canine parvovirus infection, it may be possible that the therapeutic benefits observed in this study were mediated, at least in part, by the anti-inflammatory properties of rFeIFN-ω [13,16,17,18]. Furthermore, IFN-ω has been shown to possess in vitro antiproliferative activities against various tumor cells, including feline fibrosarcoma and mammary tumor cells, as well as canine malignant melanoma and squamous carcinoma [13,22,23,25]. It can be speculated that rFeIFN-ω might also influence local cellular responses, such as modulating fibroblasts or angiogenic processes involved in hematoma cavity formation. However, because cytological, histological, or molecular evaluations were not performed in the present study, these mechanistic explanations remain purely theoretical. Dedicated in vitro and in vivo studies are necessary to rigorously evaluate the anti-inflammatory and cellular effects of rFeIFN-ω on canine auricular tissues.

In the present study, no dogs undergoing the combination therapy of local rFeIFN-ω injection and oral prednisolone exhibited clinically relevant adverse effects. However, due to the retrospective nature of this study, pre- and post-treatment laboratory evaluations (e.g., blood tests) and diagnostic imaging were rarely performed. To rigorously evaluate the systemic safety profile, objective laboratory and imaging monitoring before and after therapy should be implemented in future prospective trials. Notably, one excluded dog treated with local rFeIFN-ω 10.0 MU injection alone developed an allergic reaction characterized by facial swelling. This clinical observation suggests that concurrent oral prednisolone administration may play a role in mitigating the risk of rFeIFN-ω-induced allergic reactions [26,27]. Nevertheless, because this allergic event occurred in only a single case, the true incidence of hypersensitivity reactions secondary to local rFeIFN-ω administration remains unclear. Comprehensive investigations focusing on systemic adverse effects and the determination of a safe and optimal dosage range are essential.

The favorable clinical outcomes observed in this study may stem not only from the intrinsic properties of rFeIFN-ω but also from the concurrent anti-inflammatory action of oral prednisolone [7,11]. Furthermore, oral prednisolone is simultaneously highly effective for managing underlying conditions, such as otitis externa and allergic skin diseases, which frequently serve as the primary etiologies for the development of aural hematoma [3,28,29,30]. Given the diverse physiological activities of IFN, rFeIFN-ω may potentiate the therapeutic efficacy of prednisolone against aural hematomas. While this synergistic potential could represent a novel therapeutic strategy for other inflammatory diseases by enhancing prednisolone efficacy, it also carries a theoretical risk of exacerbating corticosteroid-related adverse effects [12]. Although no obvious adverse events were noted in our cohort, clinicians should exercise caution regarding the dosage of concurrent prednisolone when utilizing this combination therapy.

There is a lack of epidemiological studies regarding canine aural hematoma [1,2]. Based on the results of the present study, it is difficult to investigate the breed predisposition to aural hematoma. This is because the present study is retrospective and was conducted at a single, specific private animal hospital, making it impossible to perform an evaluation that excludes regional bias or numerical bias. Although French Bulldogs, Labrador Retrievers, and Golden Retrievers were relatively frequent in this cohort, the small sample size and single-center retrospective design preclude any inference regarding breed predisposition.

Several limitations should be considered when interpreting the findings of this study. First, the doses of rFeIFN-ω administered in this study were limited to either 10 MU, which were selected primarily for procedural ease of local administration and based on the commercially available product formulations. Second, due to the retrospective nature of this study utilizing medical records, we were unable to standardize the distribution of dog breeds or establish a control group. Most notably, the lack of a comparison with a prednisolone monotherapy group limits our ability to definitively attribute the observed clinical success solely to the therapeutic effects of rFeIFN-ω. Furthermore, another major limitation is the subjective nature of our clinical outcome assessment. Because this was a retrospective review of routine medical records, objective quantitative measurements, such as caliper measurements of pinna thickness, ultrasonographic fluid volume estimation, or standardized photographic scoring, were not consistently performed. Consequently, clinical outcome determinations relied on qualitative physical evaluations by attending clinicians, which may introduce observer subjectivity. Prospective randomized controlled trials are warranted to provide more robust, high-quality data regarding the efficacy of this combination therapy. Third, the detailed mechanism of action by which rFeIFN-ω exerts its therapeutic effects on aural hematomas remains to be fully elucidated. Finally, prospective and detailed safety profiles of rFeIFN-ω in canine patients are still lacking. Although rFeIFN-ω is commercially approved for feline infectious diseases, comprehensive safety studies focusing on its administration in dogs remain limited [17,18]. Although no obvious systemic adverse effects were documented in our cohort, further investigation is essential to establish the precise safety margin and optimal dosing regimen in dogs.

## 4. Conclusions

In conclusion, this retrospective study demonstrates that a combination protocol of intralesional rFeIFN-ω injection and oral prednisolone was associated with favorable initial resolution rates in dogs with aural hematoma, without requiring general anesthesia or sedation. However, given the retrospective design and absence of a control group, the independent contribution of rFeIFN-ω remains unproven. Further prospective and mechanistic studies are warranted to validate these clinical findings and optimize the treatment protocol.

## Figures and Tables

**Table 1:** Baseline characteristics of the 26 dogs.

Parameter	Category/Value	Data
Total number of dogs		26
Age (years)	Median (range)	9 (1–14)
Sex [*n* (%)]	Male (Intact/Castrated)	11 (42.3%) [6/5]
	Female (Intact/Spayed)	15 (57.7%) [7/8]
Breed [*n* (%)]	French Bulldog	5 (19.2%)
	Golden Retriever	4 (15.4%)
	Labrador Retriever	4 (15.4%)
	Other breeds * (*n* = 1)	13 (50.0%)
Otitis externa [*n* (%)]	Present	11 (42.3%)

* Other breeds include Beagle, Weimaraner, American Cocker Spaniel, Welsh Corgi, Standard Poodle, Toy Poodle, West Hiland White Terrier, Shiba Inu, Shetland Sheepdog, German Shepherd, Maltese, Miniature Pinscher, and Mixed-breed (one dog each).

**Table 2:** Clinical outcomes following the combination treatment protocol.

Clinical Parameter	Value/Rate	95% Confidence Interval (CI) *
Number of treatment sessions (means ± SD)	2.37 ± 1.14	―
Clinical improvement rate [*n*/*N* (%)]	26/26 (100%)	86.8–100.0%
Complete initial resolution rate [*n*/*N* (%)]	21/26 (80.8%)	60.7–93.5%
Recurrence rate [*n*/*N* (%)]	5/21 (23.8%)	8.2–47.2%
Adverse effects	None recorded	―

* Calculated using the Clopper-Pearson exact method.

## Data Availability

The original contributions presented in this study are included in the article/Supplementary Material. Further inquiries can be directed to the corresponding author.

## Supplementary Materials

The following supporting information can be downloaded at https://www.mdpi.com/article/10.3390/vetsci13090937/s1, Table S1: Cases and medical details.

## Abbreviation

The following abbreviation is used in this manuscript:

rFeIFN-ω	Recombinant feline interferon-omega
