# Supplementary material for: Use of the Combination Therapy of Recombinant Feline Interferon-Omega Injection and Oral Prednisolone for Canine Aural Hematoma: A Retrospective Case Series
Source: Vet Sci. 2026 Sep 10;13(9):937. doi: 10.3390/vetsci13090937 (PMC13611881; doi:10.3390/vetsci13090937)
Supplement: Supplementary file 1 [file vetsci-13-00937-s001.zip › vetsci-4517114-supplementary-Table S1 Cases and medical details.pdf]

Case Number	Breed	Sex	Age at Onset (y)	Affected Side	Tretment Sessions	Efficacy Outcome	Recurrence Status	Retreated Sessions	Presence of Concurrent Otitis Externa	Adverse Effects
1	Labrador Retriever	Female	11	Left	2	Complete Resolution	Recurred after 1 m	2	Present	Not recorded
2	French Bulldog	Male	3	Left	1	Improved	No recurrence observed	Not applicable	Present	Not recorded
3	German Shepherd	Male	4	Right	2	Complete Resolution	No recurrence observed	Not applicable	Present	Not recorded
4	Maltese	Castrated Male	5	Left	5	Complete Resolution	Recurred after 6 m	No retreated	Absent	Not recorded
5	Labrador Retriever	Spayed Female	8	Right	1	Complete Resolution	Recurred after 1 y	No retreated	Absent	Not recorded
6	Shetland Sheepdog	Female	10	Right	2	Complete Resolution	Recurred after 2 m	1	Present	Not recorded
7	Shiba Inu	Castrated Male	9	Right	2	Complete Resolution	Recurred after 2 wks	No retreated	Absent	Not recorded
8	French Bulldog	Castrated Male	9	Right	2	Complete Resolution	No recurrence observed	Not applicable	Absent	Not recorded
9	Miniature Pinscher	Castrated Male	8	Left	3	Complete Resolution	No recurrence observed	Not applicable	Absent	Not recorded
10	West Highland White Terrier	Male	14	Left	4	Complete Resolution	No recurrence observed	Not applicable	Present	Not recorded
11	French Bulldog	Castrated Male	13	Left	2	Complete Resolution	No recurrence observed	Not applicable	Present	Not recorded
12	Golden Retriever	Female	3	Left	3	Complete Resolution	No recurrence observed	Not applicable	Absent	Not recorded
13	Golden Retriever	Spayed Female	6	Right	2	Complete Resolution	No recurrence observed	Not applicable	Absent	Not recorded
14	Golden Retriever	Female	10	Right	2	Complete Resolution	No recurrence observed	Not applicable	Absent	Not recorded
15	Standard Poodle	Spayed Female	8	Right	1	Complete Resolution	No recurrence observed	Not applicable	Absent	Not recorded
16	Labrador Retriever	Spayed Female	12	Right	3	Complete Resolution	No recurrence observed	Not applicable	Present	Not recorded
17	Toy Poodle	Spayed Female	10	Left	2	Complete Resolution	No recurrence observed	Not applicable	Present	Not recorded
18	French Bulldog	Spayed Female	1	Right	2	Complete Resolution	No recurrence observed	Not applicable	Absent	Not recorded
19	Golden Retriever	Spayed Female	9	Left	1	Improved	No recurrence observed	Not applicable	Present	Not recorded
20	Labrador Retriever	Male	13	Right	3	Complete Resolution	No recurrence observed	Not applicable	Present	Not recorded
21	American Cocker Spaniel	Male	11	Right	5	Complete Resolution	No recurrence observed	Not applicable	Present	Not recorded
22	Welsh Corgi	Male	8	Left	1	Improved	No recurrence observed	Not applicable	Absent	Not recorded
23	Mixed	Female	8	Left	3	Improved	No recurrence observed	Not applicable	Absent	Not recorded
24	Weimaraner	Female	10	Left	3	Improved	No recurrence observed	Not applicable	Absent	Not recorded
25	French Bulldog	Castrated Male	9	Left	2	Complete Resolution	No recurrence observed	Not applicable	Absent	Not recorded
26	Beagle	Female	11	Unknown	4	Complete Resolution	No recurrence observed	Not applicable	Absent	Not recorded
